# Steroid Hormone Biosynthesis Metabolism Is Associated With Fatigue Related to Androgen Deprivation Therapy for Prostate Cancer

**DOI:** 10.3389/fcell.2021.642307

**Published:** 2021-05-17

**Authors:** Li Rebekah Feng, Jennifer J. Barb, Hannah Allen, Jeniece Regan, Leorey Saligan

**Affiliations:** ^1^National Institute of Nursing Research, National Institutes of Health, Bethesda, MD, United States; ^2^Clinical Center, National Institutes of Health, Bethesda, MD, United States

**Keywords:** cancer-related fatigue, radiation therapy, prostate cancer, androgen deprivation therapy, metabolomics, steroid hormone biosynthesis, androgen metabolism

## Abstract

**Background:**

Androgen deprivation therapy (ADT) is a cornerstone treatment for prostate cancer. Despite the clinical benefits, ADT is associated with multiple adverse effects including fatigue. The goal of the study was to examine metabolomic changes to better understand cancer-related fatigue specific to ADT treatment.

**Methods:**

A total of 160 plasma samples collected from participants with (+ADT, *n* = 58) or without neoadjuvant ADT (−ADT, *n* = 102) prior to radiation therapy for treatment of non-metastatic localized prostate cancer were included in the study. Fatigue and sleep-related impairment were measured using the Patient Reported Outcomes Measurement Information System. Plasma metabolites were identified and measured using untargeted ultrahigh-performance liquid chromatography/mass spectrometry metabolomics analyses. Partial least square discriminant analysis was used to identify discriminant metabolite features, and the diagnostic performance of selected classifiers was quantified using AUROC curve analysis. Pathway enrichment analysis was performed using metabolite sets enrichment analyses.

**Findings:**

Steroid hormone biosynthesis pathways, including androstenedione metabolism as well as androgen and estrogen metabolism, were overrepresented by metabolites that significantly discriminated samples in the +ADT from the −ADT group. Additional overrepresented metabolic pathways included amino acid metabolism, glutathione metabolism, and carnitine synthesis. Of the metabolites that were significantly different between the groups, steroid hormone biosynthesis metabolites were most significantly correlated with fatigue severity. Sleep-related impairment was strongly correlated with fatigue severity and inversely correlated with ADT-induced reduction in androsterone sulfate.

**Conclusions:**

Patients with non-metastatic prostate cancer receiving neoadjuvant ADT prior to radiation therapy reported relatively more severe fatigue. Increased fatigue in this population may be attributable to sleep-related impairment associated with alterations in steroid hormone biosynthesis. Findings in this study provide a basis for further research of changes in sleep patterns and their role in this specific subcategory of cancer-related fatigue caused by the treatment.

## Introduction

Androgen-deprivation therapy (ADT) is considered a cornerstone treatment option for prostate cancer, the second leading cause of cancer mortality among North American men (Duchesne et al., 2016). Nearly 50% of all patients with prostate cancer will undergo ADT at some point after diagnosis, either as primary, neoadjuvant, or secondary therapy (Alibhai et al., 2010). The effects of hormonal ADT in suppressing tumor growth and delaying metastasis was first reported in 1941 and is thought to be related to the ubiquitous expression of androgen receptors in prostate cancer and the dependence of tumor cell growth on the transcription of specific pro-survival genes downstream from androgen receptor signaling (Harris et al., 2009).

Despite the significant survival advantage it confers, ADT is associated with numerous adverse effects including increased risk for cardiovascular diseases, diabetes, sexual dysfunction, cognitive, and mood dysfunction, and sleep disturbance (Gonzalez et al., 2018; Siddiqui and Krauss, 2018). One of the most common and debilitating symptoms of both cancer and ADT is fatigue, reported by up to 80% of oncology patients and 40% of patients receiving ADT (Nelson et al., 2016). Cancer-related fatigue is among the most debilitating symptoms related to cancer or cancer treatment and a common cause for falls leading to physical disability, inability to work, and feelings of hopelessness and despair (Vande Walle et al., 2014; Wolvers et al., 2019). There is an urgent need to understand the underlying mechanisms of cancer-related fatigue to find ways to better manage this common condition (Berger et al., 2015).

Metabolomic profiling refers to the comprehensive identification and quantification of endogenous or exogenous small-molecule metabolites (Lanznaster et al., 2018). This method is particularly well-suited for biomarker discovery because the metabolomic status in a biospecimen directly reflects the chemical transformation during metabolism, depicting both the steady-state equilibrium and dynamic responses to physiological stimuli (Tolstikov et al., 2020). The targeted approach refers to quantitation of chemically annotated metabolites using isotope labeling; the untargeted metabolomics, on the other hand, is the unbiased and comprehensive approach that first detects distinct chromatographic features, such as mass-to-charge ratio, and subsequently identifies metabolites using the reference spectral library (Ribbenstedt et al., 2018). In recent years, metabolomics has gained popularity as a powerful tool for biomarker discovery and mechanistic investigations that complement other -omics methodologies, providing invaluable information on tissue specificity and temporal dynamics (Armstrong et al., 2012).

Cancer-related fatigue is a clinical condition that likely encompasses a multitude of subcategories with different pathogenic mechanisms that lead to the same subjective experience (Berger et al., 2015). For example, previous studies have shown that cancer-related fatigue that lasts up to a year may be due to unresolved inflammation (Feng et al., 2017, 2018a), whereas acute fatigue during radiation therapy with neoadjuvant ADT may be related to anemia and mitochondrial dysfunction (Feng et al., 2015, 2018b). The complexity of the symptom and the heterogeneity of underlying mechanisms make metabolomics particularly well-suited for studying cancer-related fatigue. Our goal in this study was to focus specifically on mechanisms of fatigue related to androgen deprivation in men with non-metastatic localized prostate cancer. We utilized an unbiased comprehensive approach to examine metabolic changes associated with this specific ADT-related subcategory of cancer-related fatigue. We further explored the contribution of ADT-induced sleep impairment to increased fatigue reported by patients who received ADT.

## Materials and Methods

### Study Participants

This study was approved by the National Institutes of Health (NIH) Institutional Review Board. All participants were men with confirmed diagnoses of localized non-metastatic adenocarcinoma of the prostate who were scheduled to receive external-beam radiation therapy. Exclusion criteria included progressive illnesses, psychiatric diseases within the past 5 years, uncorrected hypothyroidism, anemia, a second malignancy, and use of sedatives, steroids, or non-steroidal anti-inflammatory agents. At the time of the study, participants in the +ADT group received on average 52 days of ADT prior to starting external-beam radiation therapy. The ADT treatment included 22.5 mg leuprolide acetate, a gonadotropin-releasing hormone (GnRH) agonist, and a daily dose of 50 mg bicalutamide, an androgen receptor antagonist (Sharifi et al., 2005). Participants were recruited at the NIH Magnuson Clinical Research Center, Bethesda, MD, United States. Signed written informed consents were obtained prior to study participation.

### Instruments

Sleep quality was measured using the Patient Reported Outcomes Measurement Information System (PROMIS^TM^) v1.0 – Sleep Related Impairment (PROMIS-SRI) Short Form 8b, an eight-question form that quantifies the extent to which sleep impairment impacts daily life (Buysse et al., 2010; Yu et al., 2012). Raw scores ranging from 8 to 40 are converted to a *T*-score with a mean of 50 (Yu et al., 2012). A lower PROMIS-SRI *T*-score indicates better sleep, and a higher *T*-score indicates increased impairment due to reduced sleep (Yu et al., 2012). Fatigue was quantified with the PROMIS v1.0 – Fatigue (PROMIS-Fatigue) Short Form 7a, which measures the impact in the last 7 days that fatigue has on daily life (Ameringer et al., 2016). Raw scores range from 7 to 35, and the *T*-score ranges from 29.4 to 83.2, with a mean of 50 (Cook et al., 2012). Similar to PROMIS-SRI, higher scores on the PROMIS-Fatigue scale indicate higher fatigue symptom severity (Rothrock et al., 2010). The PROMIS *T*-score metric is anchored to the United States general population, and a cutoff *T*-score of 50 differentiates clinically meaningful fatigue in the oncology population (Cella et al., 2014).

### Metabolomics

Untargeted metabolomics analysis was performed at Metabolon, Inc., (Durham, NC, United States), as previously described (Collet et al., 2017). Briefly, individual plasma samples were subjected to methanol extraction and divided into aliquots for analysis. Several recovery standards were added prior to the first step in the extraction process for QC purposes. To remove proteins, dissociate small molecules bound to proteins, or trapped in the precipitated protein matrix, and recover chemically diverse metabolites, proteins were precipitated with methanol under vigorous shaking for 2 min (Glen Mills GenoGrinder 2000) followed by centrifugation. The resulting extract was divided into five fractions: two for analysis by two separate reverse-phase (RP)/UPLC-MS/MS methods with positive ion mode electrospray ionization (ESI), one for analysis by RP/UPLC-MS/MS with negative ion mode ESI, one for analysis by HILIC/UPLC-MS/MS with negative ion mode ESI, and one sample reserved for backup. All methods utilized a Waters ACQUITY ultra-performance liquid chromatography (UPLC) and a Thermo Scientific Q-Exactive high-resolution/accurate mass spectrometer interfaced with a heated electrospray ionization (HESI-II) source and Orbitrap mass analyzer operated at 35,000 mass resolutions. The sample extract was dried then reconstituted in solvents compatible to each of the four methods. Each reconstitution solvent contained a series of standards at fixed concentrations to ensure injection and chromatographic consistency. One aliquot was analyzed using acidic positive ion conditions, chromatographically optimized for more hydrophilic compounds. In this method, the extract was gradient eluted from a C18 column (Waters UPLC BEH C18-2.1 × 100 mm, 1.7 μm) using water and methanol, containing 0.05% perfluoropentanoic acid (PFPA) and 0.1% formic acid (FA). Another aliquot was also analyzed using acidic positive ion conditions; however, it was chromatographically optimized for more hydrophobic compounds. In this method, the extract was gradient eluted from the same aforementioned C18 column using methanol, acetonitrile, water, 0.05% PFPA, and 0.01% FA and was operated at an overall higher organic content. Another aliquot was analyzed using basic negative ion optimized conditions using a separate dedicated C18 column. The basic extracts were gradient eluted from the column using methanol and water; however, with 6.5 mM ammonium bicarbonate at pH 8. The fourth aliquot was analyzed *via* negative ionization following elution from a HILIC column (Waters UPLC BEH Amide 2.1 × 150 mm, 1.7 μm) using a gradient consisting of water and acetonitrile with 10 mM ammonium formate, pH 10.8. The MS analysis alternated between MS and data-dependent MSn scans using dynamic exclusion. The scan range varied slighted between methods but covered 70–1,000 m/z.

Raw data were extracted, peak-identified, and QC-processed using Metabolon’s hardware and software. Compounds were identified by comparison to library entries of purified standards or recurrent unknown entities. The reference library consists of authenticated standards that contain the retention time/index (RI), mass-to-charge ratio (m/z), and chromatographic data (including MS/MS spectral data) on all molecules present in the library. Furthermore, biochemical identifications are based on three criteria: retention index within a narrow RI window of the proposed identification, accurate mass match to the library ± 10 ppm, and the MS/MS forward and reverse scores between the experimental data and authentic standards. The MS/MS scores are based on a comparison of the ions present in the experimental spectrum to the ions present in the library spectrum. While there may be similarities between these molecules based on one of these factors, the use of all three data points can be utilized to distinguish and differentiate biochemicals. The QC and curation processes were designed to ensure accurate and consistent identification of true chemical entities and to remove those representing system artifacts, mis-assignments, and background noise. Library matches for each compound were checked for each sample and corrected if necessary, and peaks were quantified using the area under the curve.

### Statistical Analysis

Metabolite concentrations were normalized to sample volume utilized for extraction and rescaled to set the median equal to 1. Metabolite concentrations were subsequently interquantile range (IQR) filtered and analyzed using univariate ANOVA and *t*-tests (unpaired, unequal variance assumed) to generate the volcano plots. IQR-filtered data were further log transformed, autoscaled, and analyzed using partial least square-discriminant analysis (PLS-DA) to determine the variance importance in projection (VIP). Multiple comparisons were adjusted with the Benjamini–Hochberg false discovery rate (FDR) method (Benjamini and Hochberg, 1995). LOOCV cross-validation and permutation tests were performed to test the model with Q^2^ and R^2^ being used to assess the robustness of the model. Metabolites were considered significant features for further analysis at VIP > 1.5, | log2 fold change| > 1.5, and FDR ≤ 10% (Newell et al., 2016). Diagnostic performance of selected classifiers was quantified using the Area under the Receiver Operating Characteristics (AUROC) curve analysis. Metabolite pathway analysis metabolite set enrichment analysis (MSEA) was performed in MetaboAnalyst 4.0 as previously described (Xia and Wishart, 2016). Statistical significance was defined as *p* < 0.05. Data analyses were performed using a combination of JMP Pro^TM^ Statistical Discovery Software 15 15.0.0 (SAS Institute, Cary, NC, United States).

## Results

A total of 160 plasma samples collected from participants with (+ADT, *n* = 58) or without neoadjuvant ADT (−ADT, *n* = 102) prior to radiation therapy for treatment of non-metastatic localized prostate cancer were included in the current study (Table 1). All study participants were older men with an average of 66 ± 7.07 years of age (Table 1). Participants who had received ADT exhibited higher body mass index (BMI) as compared to those without ADT (Table 1; +ADT: 29.65 ± 4.83; −ADT: 28.00 ± 4.30). There was no statistically significant difference in education or ethnicity between the two groups (Table 1). A larger proportion of participants exhibited higher Gleason scores in the +ADT group, whereas no significant difference was observed in the *T*-stage between +ADT and −ADT groups (Table 1).

**TABLE 1:** Demographics and clinical characteristics of sample population.

	Total (*n* = 160)	ADT (*n* = 58)	No ADT (*n* = 102)	*P*-value
Age (years)	66 ± 7.07	67.31 ± 7.15	64.30 ± 8.70	0.027
BMI (kg/m2)	28.6 ± 4.56	29.65 ± 4.83	28.00 ± 4.30	<0.0001
**Race**				
African American	21.25%	24.14%	19.61%	0.751
Asian	5.00%	3.45%	5.88%	
Caucasian	72.50%	72.41%	72.55%	
Unknown	1.25%	0.00%	1.96%	
**Ethnicity**				
Hispanic/Latino	2.50%	1.70%	2.94%	0.64
Non-Hispanic/Latino	96.25%	98.30%	95.10%	
Unknown	1.25%	0.00%	1.96%	
**Education**				
Eighth grade or less	0.63%	1.72%	0.00%	0.058
High school	11.88%	12.07%	11.76%	
Vocational/Technical Degree	18.13%	8.62%	23.53%	
Associate degree/some college	5.00%	10.34%	1.96%	
Bachelor’s degree	17.50%	20.69%	15.69%	
Advanced degree	45.63%	44.83%	46.08%	
Unknown	1.25%	1.72%	0.98%	
***T*-stage**				
T0	5.00%	0.00%	7.84%	0.15
T1c	36.88%	34.48%	38.24%	
T2a-c	34.38%	34.48%	34.31%	
T3a-c	20.00%	25.86%	16.67%	
T4	3.75%	5.17%	2.94%	
**Gleason score**				
6	21.90%	0.00%	34.31%	<0.0001
7	38.10%	39.66%	37.25%	
8	25.00%	32.76%	20.59%	
9	13.10%	24.14%	6.86%	
10	1.90%	3.45%	0.98%	

*BMI, body mass index; ADT, androgen deprivation therapy.*

Using untargeted LC/MS, we found 1,120 compounds of known identity. After applying an interquartile range filter, a total of 661 metabolites remained: 315 lipids, 140 xenobiotics, 120 amino acids, 27 cofactors and vitamins, 13 carbohydrates, 13 nucleotides, 12 peptides, and 18 partially characterized molecules.

**FIGURE 1:**
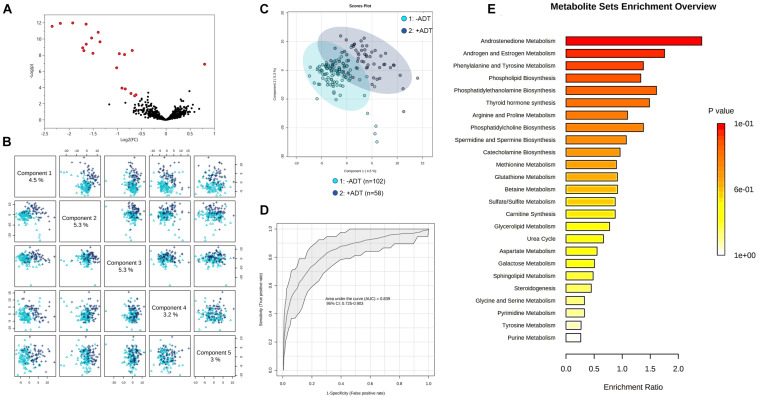
Metabolic profiles of patients with non-metastatic prostate cancer with (+ADT, *n* = 58) or without androgen deprivation therapy (−ADT, *n* = 102). **(A)** Volcano plot of metabolites of the +ADT group compared to −ADT. The y axis represents *p*-value converted to negative log 10 scale, and the x axis represents log2 fold change. Significant metabolites (fold change > 1.5, FDR ≤ 0.1) were highlighted in red. **(B)** Pairwise PLSDA score plot of the top five components. **(C)** PLSDA two-dimensional plot ellipses representing 95% confidence intervals. **(D)** ROC curve demonstrating the specificity and sensitivity of the PLSDA model discriminating the +ADT group from the −ADT group. AUC = 0.839, 95% CI (0.725, 0.903). Excellent classification is indicated by an AUC > 0.90. **(E)** Metabolite set enrichment analysis (MSEA) of significant metabolites.

**FIGURE 2:**
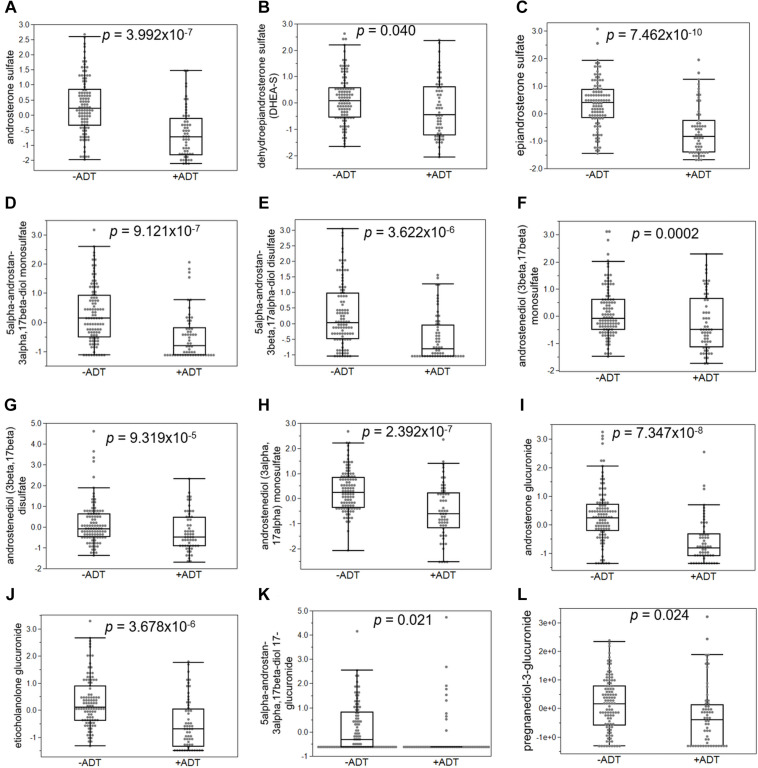
Androgen deprivation therapy broadly affected metabolites related to steroid hormone biosynthesis. **(A)** Androsterone sulfate was significantly decreased in patients with ADT. Sulfated **(B–H)** and glucuronidated androgen metabolites **(I–L)** were significantly decreased in the +ADT group. *p < 0.05 and false-discovery rate ≤ 10%.

**FIGURE 3:**
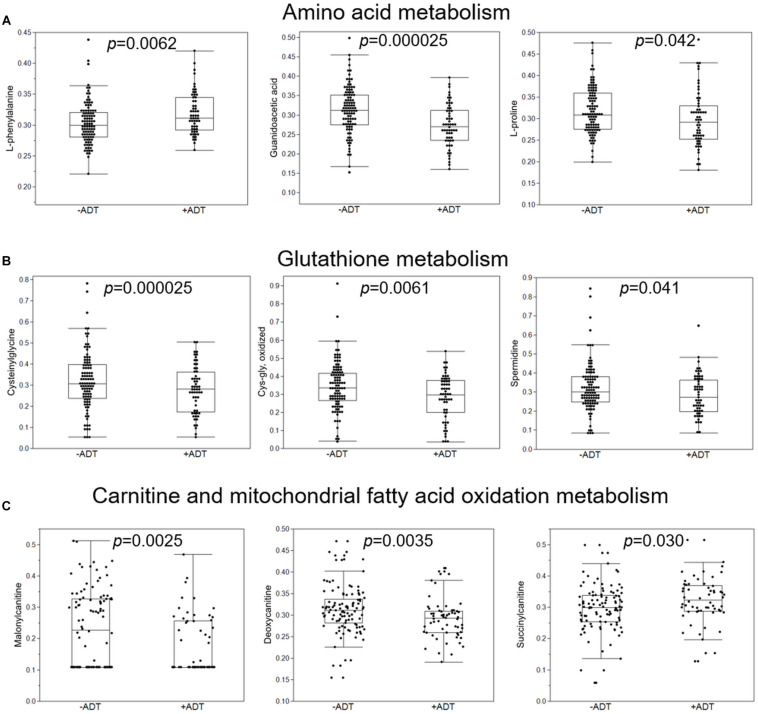
Additional metabolic pathways that were overrepresented by metabolites significantly distinguished the +ADT from the −ADT group. **(A)** Box plots of individual metabolites related to amino acid metabolism. **(B)** Box plots of individual metabolites related to glutathione metabolism. **(C)** Box plots of individual metabolites related to carnitine and mitochondrial fatty acid oxidation metabolism.

First, we compared the overall metabolomic profiles of the +ADT vs. −ADT groups. A total of 28 metabolites were found to be significant features for further analyses based on a log2 fold change cutoff of 1.5 at 10% false-discovery rate (Figure 1A; see Table 2 for detailed chemical information), and a PLSDA VIP score of ≥1.5 (Figures 1B,C). The receiver operator characteristic curve analysis (AUROC) demonstrated good discriminating power of the classification model to distinguish the +ADT from the −ADT group [Figure 1D: AUC = 0.839, 95% CI (0.725, 0.903)]. As expected for the intended effect of ADT, metabolite set enrichment analysis (MSEA) of the significantly different metabolites revealed steroid hormone biosynthesis pathways, including androstenedione metabolism as well as androgen and estrogen metabolism, to be overrepresented by metabolites that were significantly different between +ADT and −ADT groups (Figure 1E). Box plots of individual metabolites related to steroid hormone biosynthesis are shown in Figure 2. Sulfated (Figures 2A–H) as well as the glucuronide steroid hormone metabolic products (Figures 2I–L) were decreased by ADT, demonstrating the effectiveness of ADT. Although the length of treatment in the +ADT group varied [mean = 52.53 days, 95% CI (44.65, 60.43)], reductions in androsterone sulfate levels were not significantly correlated with the length of ADT treatment (*p* = 0.10). Notably, the various degrees to which ADT decreased levels of androsterone sulfate illustrated the heterogeneity of the response to treatment in the +ADT group (Figure 2A). Additional top enriched pathways (Figure 1E) also included amino acid metabolism pathways (Figure 3A), glutathione metabolism (Figure 3B), and carnitine synthesis (Figure 3C).

**TABLE 2:** Chemical information of metabolites of interest.

Metabolite ID	Super pathway	Sub pathway	Compound ID	Platform	Chemical ID	RI (retention time/index)	Mass/Charge ratio
epiandrosterone sulfate	Lipid	Androgenic Steroids	33973	LC/MS Neg	100001287	4855	369.1741
androsterone glucuronide	Lipid	Androgenic Steroids	61846	LC/MS Neg	100002761	4953	465.2494
androsterone sulfate	Lipid	Androgenic Steroids	31591	LC/MS Neg	100001073	5022	369.1741
5alpha-androstan-3alpha,17beta-diol monosulfate (2)	Lipid	Androgenic Steroids	37185	LC/MS Neg	100006005	5080	371.1898
5alpha-androstan-3beta,17alpha-diol disulfate	Lipid	Androgenic Steroids	37187	LC/MS Neg	100002021	4215	225.0697
etiocholanolone glucuronide	Lipid	Androgenic Steroids	47112	LC/MS Neg	100005403	4915	465.2494
androstenediol (3alpha, 17alpha) monosulfate	Lipid	Androgenic Steroids	37207	LC/MS Neg	100002026	4712	369.1741
androstenediol (3beta,17beta) disulfate	Lipid	Androgenic Steroids	37203	LC/MS Neg	100001994	4065	224.0624
androstenediol (3beta,17beta) monosulfate	Lipid	Androgenic Steroids	37210	LC/MS Neg	100002029	4500	369.1741
N,N,N-trimethyl-5-aminovalerate	Amino Acid	Lysine Metabolism	57687	LC/MS Pos Early	100015962	2186	160.1332
glycosyl-N-(2-hydroxynervonoyl)-sphingosine (d18:1/24:1(2OH))	Lipid	Hexosylceramides (HCER)	57444	LC/MS Pos Late	100015752	3839	826.6767
behenoyldihydrosphingomyelin (d18:0/22:0)	Lipid	Dihydrosphingomyelins	57331	LC/MS Pos Late	100009026	3150	789.6844
malonylcarnitine	Lipid	Fatty Acid Synthesis	37059	LC/MS Pos Early	100001526	2086	248.1129
N-methylproline	Amino Acid	Urea cycle; Arginine and Proline Metabolism	37431	LC/MS Pos Early	100001956	1335	130.0863
sphingomyelin (d18:0/20:0, d16:0/22:0)	Lipid	Dihydrosphingomyelins	57476	LC/MS Pos Late	100015786	2600	761.6531
cys-gly, oxidized	Amino Acid	Glutathione Metabolism	18368	LC/MS Neg	1224	925	353.0595
homostachydrine	Xenobiotics	Food Component/Plant	33009	LC/MS Pos Early	100001550	1750	158.1176
chiro-inositol	Lipid	Inositol Metabolism	37112	LC/MS Polar	100001859	3191.2	225.0616
5alpha-androstan-3alpha,17beta-diol 17-glucuronide	Lipid	Androgenic Steroids	47132	LC/MS Neg	100005396	4930	467.265
pregnanediol-3-glucuronide	Lipid	Progestin Steroids	40708	LC/MS Neg	100003470	5145	495.2963
dehydroepiandrosterone sulfate (DHEA-S)	Lipid	Androgenic Steroids	32425	LC/MS Neg	100000792	4745	367.1585
spermidine	Amino Acid	Polyamine Metabolism	485	LC/MS Pos Late	50	700	146.1652
cysteinylglycine	Amino Acid	Glutathione Metabolism	35637	LC/MS Pos Early	278	2132	179.0485
phenylalanine	Amino Acid	Phenylalanine Metabolism	64	LC/MS Pos Early	460	2878	166.0863
guanidinoacetate	Amino Acid	Creatine Metabolism	43802	LC/MS Polar	344	2884	116.0466
proline	Amino Acid	Urea cycle; Arginine and Proline Metabolism	1898	LC/MS Pos Early	480	1603	116.0706
deoxycarnitine	Lipid	Carnitine Metabolism	36747	LC/MS Pos Early	100001662	2052	146.1176
succinylcarnitine (C4-DC)	Energy	TCA Cycle	37058	LC/MS PosEarly	100001948	2291	262.1285

**FIGURE 4:**
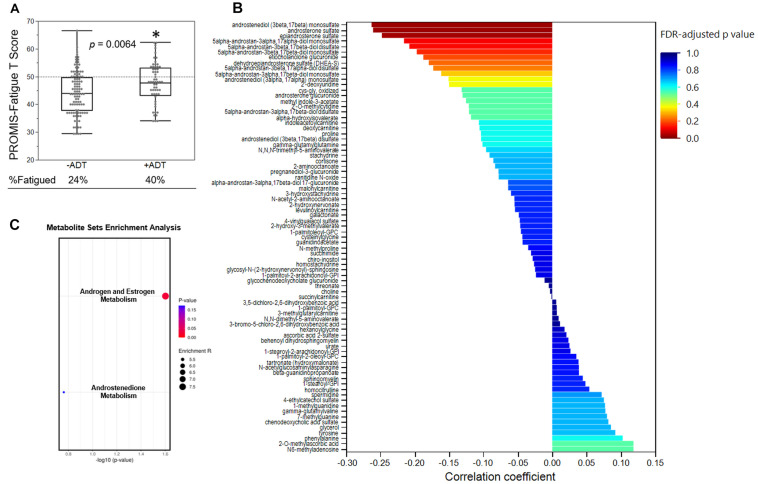
Steroid hormone biosynthesis was associated with fatigue severity. **(A)** Box plot showing the PROMIS-fatigue *T*-scores of the −ADT PROMIS-Fatigue *T*-scores of the −ADT group (44.34 ± 7.81) and the +ADT group (47.92 ± 7.28). *Indicates statistical significance (*p* = 0.0064). Scores above the dotted lines are considered fatigued (24% of −ADT, 40% of +ADT). **(B)** Correlations of PROMIS-Fatigue *T*-score and metabolites that was significantly different between the two groups. X axis indicates the correlation coefficient. Colors of the bars indicate FDR-adjusted *p*-values. **(C)** Metabolite set enrichment analysis of metabolites that significantly correlated with PROMIS-Fatigue *T*-scores. Steroid hormone biosynthesis (KEGG ID: M00107), including androgen and estrogen metabolism (SMPDB ID: SMP0000068) as well as androstenedione metabolism (SMPDB ID: SMP0030406), were significantly overrepresented.

**FIGURE 5:**
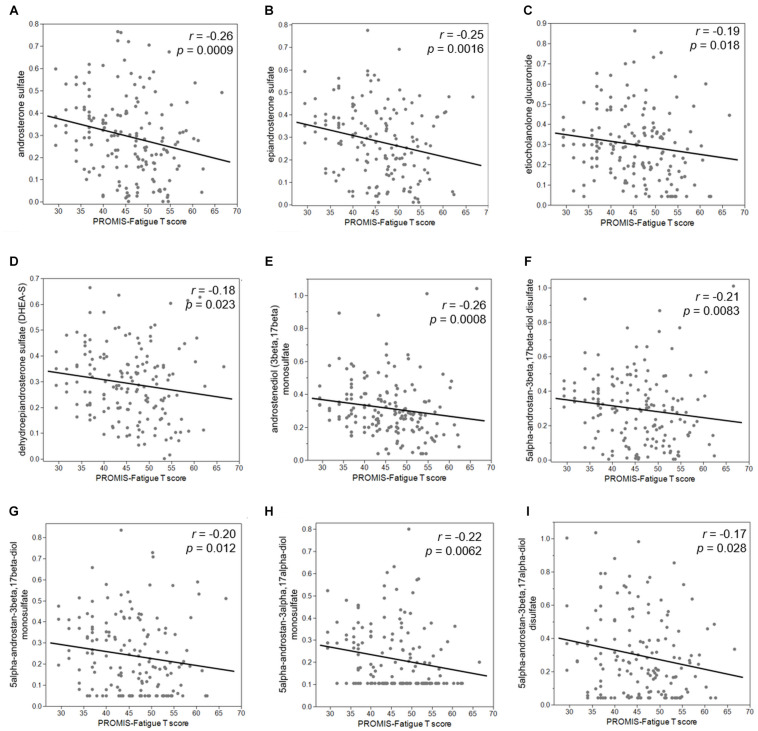
Reduced steroid hormone biosynthesis metabolites were associated with increased severity of cancer-related fatigue. PROMIS-Fatigue *T*-scores significantly correlated with major androgen metabolites including **(A)** androsterone sulfate (*r* = –0.26, *p* = 0.0009), **(B)** epiandrosterone sulfate (*r* = –0.25, *p* = 0.0016), **(C)** etiocholanolone glucuronide (*r* = –0.19, *p* = 0.018), and **(D)** dehydroepiandrosterone sulfate (DHEA-S) (*r* = –0.18, *p* = 0.023). PROMIS-Fatigue *T*-scores also correlated with sulfated metabolites of androgen including **(E)** androstenediol (3beta,17beta) monosulfate (*r* = –0.26, *p* = 0.0008), **(F)** 5alpha-androstan-3beta,17beta-diol disulfate (*r* = –0.21, *p* = 0.0083), **(G)** 5alpha-androstan-3beta,17beta-diol monosulfate (*r* = –0.20, *p* = 0.012), **(H)** 5alpha-androstan-3alpha,17alpha-diol monosulfate (*r* = –0.22, *p* = 0.0062), and **(I)** 5alpha-androstan-3beta,17alpha-diol disulfate (*r* = –0.17, *p* = 0.028).

Second, we examined the associations between PROMIS-Fatigue *T*-scores and metabolites that significantly distinguished the +ADT group from the −ADT group. A PROMIS *T*-score of 50 best differentiates clinically meaningful fatigue in the oncology population (Cella et al., 2014). Fatigue (*T*-score ≥ 50) was reported by 40% of participants in the +ADT group (PROMIS *T*-score: 47.92 ± 7.28) compared to 24% of the −ADT group (PROMIS *T*-score: 44.34 ± 7.81) (Figure 4A, *p* = 0.0064). Correlations of fatigue severity and metabolites that significantly distinguished the two groups are shown in Figure 4B. Metabolites that significantly correlated with PROMIS-Fatigue *T*-scores were overrepresented by the steroid hormone biosynthesis pathway (KEGG ID: M00107), including androgen and estrogen metabolism (SMPDB ID: SMP0000068) and androstenedione metabolism (SMPDB ID: SMP0030406) (Figure 4C), which included androsterone sulfate (*r* = −0.26, *p* = 0.0009), epiandrosterone sulfate (*r* = −0.25, *p* = 0.0016), etiocholanolone glucuronide (*r* = −0.19, *p* = 0.018), and dehydroepiandrosterone sulfate (DHEA-S) (*r* = −0.18, *p* = 0.023). PROMIS-Fatigue *T*-scores were also correlated with sulfated metabolites of androgen including androstenediol (3beta,17beta) monosulfate (*r* = −0.26, *p* = 0.0008), 5alpha-androstan-3beta,17beta-diol disulfate (*r* = −0.21, *p* = 0.0083), 5alpha-androstan-3beta,17beta-diol monosulfate (*r* = −0.20, *p* = 0.012), 5alpha-androstan-3alpha,17alpha-diol monosulfate (*r* = −0.22, *p* = 0.0062), and 5alpha-androstan-3beta,17alpha-diol disulfate (*r* = −0.17, *p* = 0.028) (Figure 5).

**FIGURE 6:**
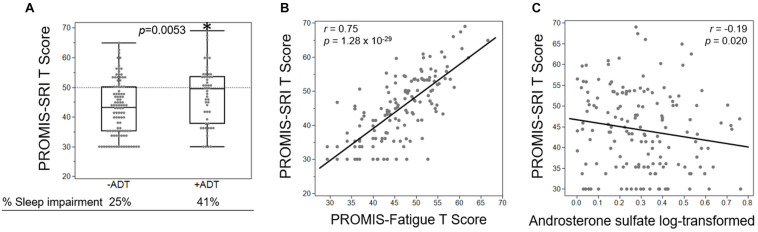
Fatigue severity was associated with ADT-related increase in sleep impairment. **(A)** Box plot showing the PROMIS-SRI *T* scores of the −ADT PROMIS-Fatigue *T*-scores of the −ADT group (42.67 ± 9.24) and the +ADT group (47.2 ± 9.95). *Indicates statistical significance (*p* = 0.0053). Scores above the dotted lines are considered fatigued (25% of −ADT, 41% of +ADT). **(B)** PROMIS-SRI *T*-score was highly correlated with PROMIS-Fatigue *T*-score (*r* = 0.75, *p* = 1.28 × 10^– 29^). **(C)** Androsterone sulfate levels significantly correlated with PROMIS-SRI *T*-scores (*r* = –0.19, *p* = 0.020).

Third, we investigated whether the increased fatigue in the +ADT group was related to sleep impairment, measured by PROMIS-SRI. Interestingly, participants in the +ADT group also reported higher levels of sleep-related impairment at 41% (*T*-score ≥ 50), compared to 25% of the −ADT group (Figure 6A, *p* = 0.0053). Self-reported sleep-related impairment (PROMIS-SRI *T*-score) was significantly correlated with cancer-related fatigue (Figure 6B, *r* = 0.75, *p* = 1.28 × 10^–29^). Of all 28 metabolites that were significantly different between +ADT and −ADT groups, only androsterone sulfate levels significantly correlated with PROMIS-SRI sleep impairment *T*-scores (Figure 6C, *r* = −0.19, *p* = 0.020), which serves as a metabolic endpoint indicator of the effectiveness of ADT in reducing circulating androgens (Chi et al., 2020).

## Discussion

**FIGURE 7:**
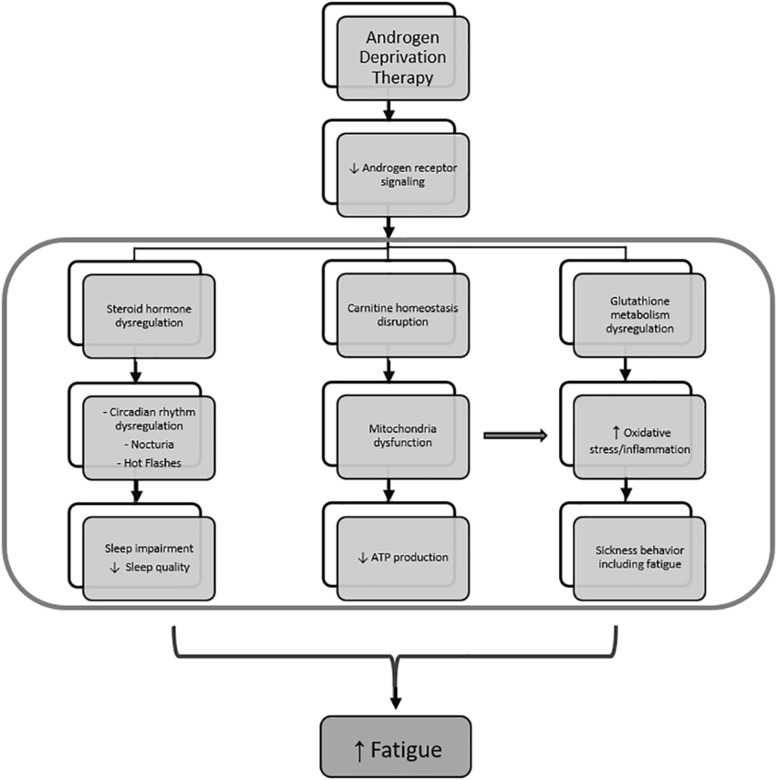
Mechanism of fatigue related to androgen deprivation therapy. Androgen deprivation therapy, which inhibits androgen receptor signaling, results in steroid hormone metabolism dysregulation and leads to sleep impairment by affecting circadian rhythm regulation, nocturia, and hot flashes. At the same time, androgen deprivation also results in dysregulated carnitine homeostasis and glutathione metabolism, leading to mitochondrial dysfunction and oxidative stress, respectively. Mitochondrial dysfunction further increase oxidative stress and contributes to inflammation-induced sickness behavior that includes fatigue.

Our goal in this study was to focus on mechanisms of a subcategory of cancer-related fatigue specifically related to ADT in patients with non-metastatic localized prostate adenocarcinoma. We examined metabolomic profile changes that may explain increased fatigue severity in response to receiving ADT (Figure 7). We found that metabolites related to steroid hormone biosynthesis best correlated with self-reported fatigue severity, which may be explained by sleep-related impairment as a result of ADT. To our knowledge, this is the first study that used the unbiased comprehensive metabolome profiling to examine the underlying mechanisms of ADT-induced increase in fatigue in men with non-metastatic prostate cancer.

Previous work showed that patients receiving ADT reported significant sleep disturbances manifested as difficulties with falling asleep and maintenance of sleep due to nocturia and hot flashes (Gonzalez et al., 2018). This is consistent with our findings that prostate cancer patients receiving ADT reported higher incidences of fatigue and sleep impairment. Of all metabolites that significantly distinguished the two groups, metabolites enriched in androstenedione metabolism as well as androgen and estrogen metabolism pathways most significantly correlated with PROMIS-Fatigue *T*-scores. The strong association between sleep-related impairment and self-reported fatigue severity suggests that the increased fatigue in the +ADT group may be due to sleep dysfunction. Importantly, of all the metabolites that significantly distinguished +ADT from −ADT group, the only metabolite that significantly correlated with PROMIS-SRI sleep impairment *T*-scores was androsterone sulfate, which is a metabolic endpoint marker of the effectiveness of ADT in reducing circulating androgens (Chi et al., 2020). This finding suggests that cancer-related fatigue specific to non-metastatic cancer patients undergoing ADT may be related to sleep impairment caused by the treatment itself. Besides physical discomfort caused by hot flashes and nocturia, the exact mechanism by which ADT causes sleep dysfunction is poorly understood (Gonzalez et al., 2018). Interestingly, androgen receptors are abundant in the suprachiasmatic nucleus (SCN) and play an important role in the neuroendocrine modulation of circadian rhythm (Mong et al., 2011). It is possible that ADT may cause sleep dysfunction, and subsequently fatigue, *via* alterations in the androgenic action on SCN circuitry and circadian rhythmicity. Although it is beyond the scope of this study, future research is needed to explore specific changes in sleep patterns caused by reduced androgen metabolites using more precise tools for measuring sleep, such as polysomnography and/or actigraphy.

Related to the natural history nature of the study design, the length of ADT treatment varied among participants in the +ADT group. Since reductions in levels of androsterone sulfate did not appear to depend on the length of treatment in the +ADT group, it is possible that the wide distribution of androsterone sulfate concentrations in the +ADT group reflected the heterogeneity in individual responses to hormonal ADT, rather than variability in treatment itself. Therefore, in analyses regarding fatigue and sleep impairment, we chose to use levels of androsterone sulfate, an indicator for the effectiveness of ADT (Chi et al., 2020), instead of a binary classification (+ADT vs. −ADT), to take into account the individual metabolic response to hormonal androgen suppression. In addition to steroid hormone biosynthesis pathways, we found preliminary indications that ADT may also affect metabolites related to carnitine homeostasis (Figures 1E, 3). Interestingly, carnitine is important for the shuttling long-chain fatty acids across the mitochondrial inner membrane and β-oxidation (Longo et al., 2016). In fact, mitochondrial dysfunction is often secondary to a disruption of carnitine homeostasis (Sharma and Black, 2009). ADT-induced changes in metabolites related to carnitine synthesis and mitochondrial fatty acid oxidation may help explain our previous observation of mitochondrial dysfunction in fatigued patients (Feng et al., 2020). Another pathway of interest that was associated with metabolites that significantly discriminated between the two groups was glutathione metabolism (Figure 1E), including cysteinylglycine and oxidized cys-gly (Figure 3B). Cysteinylglycine (cys-gly) is produced from the hydrolysis of glutathione (glutamyl-cysteinyl-glycine), one of the most important endogenous free radical scavengers (Garibotto et al., 2003). Both cysteinylglycine and oxidized cys-gly are indicators of the redox state, which can be influenced by androgen receptor signaling (Chettimada et al., 2018; Cruz-Topete et al., 2020). Lastly, androgen signaling has been shown to affect amino acid metabolism (Putluri et al., 2011; Saylor et al., 2012; Chi et al., 2020). For example, certain oncogenic mutations result in a preference for particular amino acids; the metabolic microenvironment, in turn, helps shape the genetic landscape of the tumor (Tang et al., 2015; Chi et al., 2020), although less clear is the role of the specific amino acid metabolites in fatigue pathogenesis in the +ADT group (Figures 1E, 3A). It is possible that dysregulation in both oxidative stress response and mitochondria fatty acid trafficking may affect sleep and the consequent fatigue in these patients.

One caveat of the study is that the groups were not matched in sample size and lacked in randomization. This is because the participants were part of an exploratory prospective study instead of a clinical trial and treatment decisions were made by the patients in collaboration with their oncologists. Future studies with a larger sample size, particularly in the +ADT group, will be needed to validate findings in this study. Participants in the +ADT group exhibited higher BMI compared to the −ADT group (Table 1). However, based on our unpublished data, there was no significant daily physical activity difference between the groups (*p* = 0.22, unpublished Actigraphy data), suggesting that the difference in BMI was not attributable to lifestyle differences between the groups. Although previous work has demonstrated an association between BMI and plasma levels of steroid hormones, future studies will employ more accurate measures of lean/fat mass, such as DEXA scan, to further examine the correlation between obesity and fatigue (He et al., 2018). In addition, the untargeted approach was chosen to allow for the simultaneous measurement of as many metabolites as possible to map out the ADT-related global metabolomic profile without requiring any *a priori* hypotheses. While different experimental platforms were used in this study to ensure optimized detection coverage (total number of detected metabolites of known identity: 1,120; total number of detected steroids: 42), future studies are needed to examine steroid hormones more closely using more targeted analyses. Relatedly, the pathway “androgen and estrogen metabolism” (pathway ID: SMP0000068) is an annotated metabolic pathway identified using pathway analysis (Metabolite Sets Enrichment Analysis), performed to discover biologically meaningful patterns within the data. However, the statistical significance of this pathway was likely attributable to androgen metabolites, as we did not see any difference in estrogen metabolites. Since the goal of the current study was to profile ADT-related metabolomic changes, the untargeted approach was preferable and allowed for a more global metabolomic profiling. However, it is possible the global untargeted approach is not sufficiently sensitive for estrogen measurements, particularly in male study participants. We plan to further examine androgen and estrogen metabolites in future studies using a more targeted approach. In addition, sleep impairment was measured by PROMIS-SRI, a self-report questionnaire. Future studies will be needed to assess specific changes in sleep patterns caused by ADT. Furthermore, we included participants with non-metastatic localized prostate cancer receiving neoadjuvant ADT prior to radiation therapy. Future studies will also investigate the effects of ADT as a primary therapy for treatment of advanced prostate cancer. Additionally, the effects of ADT were examined by cross-sectional comparisons in this study at one timepoint. As we continue to follow these patients, post−ADT samples will be collected to allow for measurements of longitudinal changes in hormone levels before and after ADT completion. Finally, we did not detect any significant difference between the two groups in levels of inflammatory metabolites, such as prostaglandin (*p* = 0.78) and leukotriene (*p* = 0.39). While the specific role of inflammation is beyond the scope of the current study, we hope to continue to follow these patients carefully examine the role of inflammation in this ongoing clinical protocol.

## Conclusion

In conclusion, we found that patients with non-metastatic prostate cancer receiving neoadjuvant ADT prior to radiation therapy reported increased fatigue severity compared to those without ADT. Cancer-related fatigue in patients receiving ADT may be specifically attributable to sleep-related impairment related to alterations in steroid hormone biosynthesis. These findings provide a basis for further research of changes in sleep patterns and their role in the specific subcategory of cancer-related fatigue caused by alterations in steroid hormones as a result of the treatment. Although ADT is considered an effective therapy that confers survival advantage, undesirable side effects should be taken into consideration when designing the optimal treatment strategy. As individuals may place different values on different treatment-related toxicities, knowledge of anticipated adverse effects is vitally important in designing individualized treatment plans (Loblaw et al., 2004). It is our hope that findings in this study will help patients and clinicians make more precise cost/benefit analyses when considering incorporating ADT into the treatment plan. Finally, mechanistic investigations aimed at understanding the heterogeneous pathogenic origins of cancer-related fatigue will help clinicians devise personalized and evidence-based treatment strategies.

## Data Availability Statement

All metabolomics data for “Steroid hormone biosynthesis metabolism is associated with fatigue related to androgen deprivation therapy for prostate cancer” was deposited in the Open Science Framework database (https://osf.io/) under the DOI number 10.17605/OSF.IO/SVEQK.

## Ethics Statement

The studies involving human participants were reviewed and approved by National Institutes of Health (NIH) Institutional Review Board. The patients/participants provided their written informed consent to participate in this study.

## Author Contributions

LF and LS designed the study. LF, JB, HA, JR, and LS contributed to writing and editing of the draft. LF and JB contributed to data analysis. All authors edited and approved the final draft.

## Conflict of Interest

The authors declare that the research was conducted in the absence of any commercial or financial relationships that could be construed as a potential conflict of interest.
